# Surface Electromyographic Features for Severity Classification in Facial Palsy: Insights from a German Cohort and Implications for Future Biofeedback Use

**DOI:** 10.3390/s25092949

**Published:** 2025-05-07

**Authors:** Ibrahim Manzoor, Aryana Popescu, Alexia Stark, Mykola Gorbachuk, Aldo Spolaore, Marcos Tatagiba, Georgios Naros, Kathrin Machetanz

**Affiliations:** Department of Neurosurgery and Neurotechnology, Eberhard Karls University, Hoppe-Seyler-Straße 3, 72076 Tuebingen, Germany

**Keywords:** facial palsy, recovery, rehabilitation, grading, electromyography, biofeedback, time series features

## Abstract

**Highlights:**

**What are the main findings?**

- Facial surface EMG can assess facial palsy severity.
- Biofeedback in facial palsy can be facilitated by appropriate EMG parameters.

**What are the implications of the main findings?**

- Motion classification (movement vs. rest) by sEMG is feasible even in severe cases of facial palsy.
- The results constitute the foundation for further studies on biofeedback algorithms needed for EMG biofeedback in facial palsy.

**Abstract:**

Facial palsy (FP) significantly impacts patients’ quality of life. The accurate classification of FP severity is crucial for personalized treatment planning. Additionally, electromyographic (EMG)-based biofeedback shows promising results in improving recovery outcomes. This prospective study aims to identify EMG time series features that can both classify FP and facilitate biofeedback. Therefore, it investigated surface EMG in FP patients and healthy controls during three different facial movements. Repeated-measures ANOVAs (rmANOVA) were conducted to examine the effects of MOTION (move/rest), SIDE (healthy/lesioned) and the House–Brackmann score (HB), across 20 distinct EMG parameters. Correlation analysis was performed between HB and the asymmetry index of EMG parameters, complemented by Fisher score calculations to assess feature relevance in distinguishing between HB levels. Overall, 55 subjects (51.2 ± 14.73 years, 35 female) were included in the study. RmANOVAs revealed a highly significant effect of MOTION across almost all movement types (*p* < 0.001). Integrating the findings from rmANOVA, the correlation analysis and Fisher score analysis, at least 5/20 EMG parameters were determined to be robust indicators for assessing the degree of paresis and guiding biofeedback. This study demonstrates that EMG can reliably determine severity and guide effective biofeedback in FP, and in severe cases. Our findings support the integration of EMG into personalized rehabilitation strategies. However, further studies are mandatory to improve recovery outcomes.

## 1. Introduction

The manifestation of facial palsy (FP) can cause a significant reduction in quality of life (QoL) [1,2,3,4,5]. In addition to aesthetics and psychological problems, physical complaints such as oral incompetence, incomplete eye closure or temporomandibular joint dysfunction affect patients’ daily lives. Whereas idiopathic FP patients often recover very well, the recovery phase after iatrogenic FP can sometimes be significantly delayed [6,7]. In order to ensure a proper recovery process and therapy monitoring, it is essential to objectively quantify the degree of FP. Most commonly used conventional grading scales, such as the House–Brackmann (HB) score and the Sunnybrook scale, have the disadvantage that they are highly dependent on the examiner [8,9,10,11]. An invasive electromyography (iEMG), on the other hand, can objectively measure muscle activity, but is painful for the patient and involves considerable time and effort in everyday practice.

In this regard, surface electromyography (sEMG) may be a suitable method for quantifying the degree of FP. Surface EMG does not examine individual muscle fibers, but a summation of the electrical activity of multiple motor units, including those from neighboring muscles. This can lead to interference phenomena and potential artifacts. Despite this imprecision compared to the iEMG, a few studies have demonstrated adequate application of sEMG for FP grading [12,13,14,15,16]. Franz et al. [13] investigated the correlation between sEMG and the Sunnybrook scale using 33 patients with FP after vestibular schwannoma (VS) surgery. They found a significantly higher variability of sEMG on the lesioned side as well as a significant correlation with the Sunnybrook score. Another study developed a semi-automated assessment system for facial nerve function based on sEMG and machine learning to replace the subjective HB score with a more objective method [12]. The sEMG showed promising classification performance indicating an alternative to the HB score, especially in the assessment of facial nerve function after VS surgery. However, the underlying data are based on a small patient cohort, and the grading of EMG characteristics remains unclear due to the use of machine learning methods.

At the same time, there is evidence that sEMG applications can be used for biofeedback training in FP patients or after facial nerve reconstruction [17,18,19,20,21,22,23,24]. For instance, it has been shown that synkinetic activity can be significantly reduced or prevented by sEMG biofeedback in cases of a facial aberrant reinnervation syndrome (FARS) [17,24]. Cronin et al. [20] revealed that neuromuscular facial retraining in combination with EMG can significantly improve facial function, symmetry and movement even in long-term FP. However, the biofeedback training programs performed in the studies differ considerably in terms of the selected patient population, the frequency and timing of the training. In addition, for the assessment of a sEMG, multiple (time series) features (e.g., root mean square [RMS], mean absolute value [MAV], and variance [VAR]) can be surveyed [25,26,27]. Previous studies have mostly used commercial biofeedback systems designed for use on extremity muscles applying mainly the MAV, RMS and integrated EMG (iEMG) [28].

Nevertheless, it is still quite unclear which parameters are best suited for facial applications and can be used for monitoring the degree of FP on the one hand and for biofeedback training on the other. The aim of this study was to identify sEMG time series parameters that are suitable for the classification and biofeedback of FP.

## 2. Materials and Methods

### 2.1. Study Cohort

This prospective, single center study examined a total of 55 German subjects with facial sEMG to find suitable sEMG parameters both for detecting the degree of FP and for facial biofeedback. Patients were recruited during in- and outpatient stays at the Department of Neurosurgery at the University Hospital Tübingen, Germany, between July and December 2024. They had either undergone previous tumor resection in the cerebellopontine angle or were diagnosed with idiopathic facial palsy, and presented with or without unilateral facial palsy of varying severity according to the HB score. In addition, a healthy cohort (without cranial surgery) was also recruited at the Tübingen University Hospital. Exclusion criteria were bilateral facial palsy, cognitive deficits, pregnancy and factors that make EMG measurement in the face impossible (e.g., skin rash, heavy beard growth). The study was approved by the local ethics committee and performed in accordance with the Declaration of Helsinki. All participants gave written informed consent.

### 2.2. Data Acquisition and Experimental Setup

**Figure 1:**
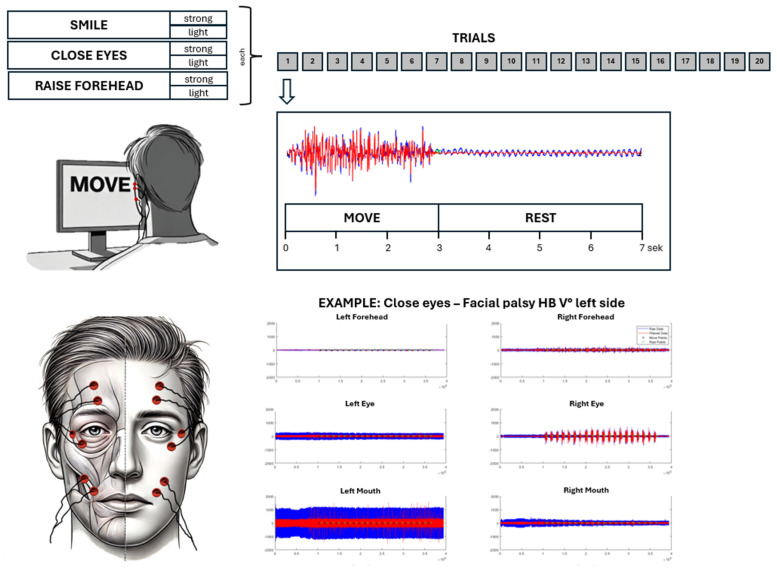
Experimental setup. Six EMG channels are recorded according to the shown template (bottom left) during the performance of six mimic movement exercises/runs (top left). Each run consisted of 20 trials, which were composed of a MOVE (3 s) and a REST interval (4 s) (top right).

After grading the facial palsy according to the HB score (I: no facial palsy to VI: complete paralysis) and positioning the electrodes (reusable Ag-AgCl snap electrodes with 4 mm diameter, Biopac Systems, Inc., Goleta, CA, USA) in a bipolar setting according to the scheme in Figure 1, each participant took part in a 30 min sEMG session. A bipolar electrode configuration was selected, as previous studies indicated a potential advantage in surface EMG applications in comparison to monopolar and common average reference configurations [29]. Furthermore, special consideration was given to the impedances being as small as feasible in order to achieve the best possible EMG signal quality [30]. The session consisted of 6 runs with different movements, which were performed in a randomized order: smile strongly, smile lightly, close eyes strongly, close eyes lightly, raise forehead strongly, raise forehead lightly. Each run consisted of 20 trials, i.e., the same movement sequence was performed 20 times with a 3 s MOVE interval and a 4 s REST interval. The patients received the commands via a computer screen in front of them. In addition, they received an auditory signal at the beginning of the REST cycle. Prior to starting the experiment, subjects were instructed in detail about the meaning of the commands. During “smile strongly”, they were supposed to smile with maximum force, while during “smile lightly”, they were asked to smile with a slight mouth angle excursion. Closing the eyes meant always closing the eyes completely, but squinting hard during “close eyes strongly” and only closing them gently during “close eyes lightly”. Finally, “raise forehead strongly” meant raising the forehead again with maximum force, while the forehead should only be raised a little during “raise forehead lightly”. Throughout the whole session, a trained investigator was present in the room and monitored the performance of each trial to ensure that all commands were understood and executed correctly. The entire EMG dataset was streamed with the commercial Neuro Omega (software version 1.6.5.0) recording device (Alpha Omega Engineering, Nof HaGalil, Israel) at a sampling rate of 2000 Hz and transferred and stored on an external computer.

### 2.3. EMG Analysis and Feature Extraction

Offline data analysis was performed using custom-written scripts in MATLAB (MathWorks Inc., Natick, MA, USA, R2022b). Raw electromyographic data of all movement types/runs were imported into MATLAB. Subsequently, data were filtered with a 4th order Butterworth bandpass filter (10–250 Hz) and a 4th order Butterworth bandstop filter (suppression of frequencies from 48.5–51.5 Hz). Based on the predefined timing structure of the experimental protocol (3-s MOVE, 4-s REST), the data were algorithmically segmented into REST and MOVE intervals. This ensured consistent alignment with the stimuli presented during acquisition. Following segmentation, 20 different time series features were determined for each individual interval for both the healthy and lesioned facial side. Detailed information on the sEMG features analyzed can be found in Appendix A. For further analysis, the absolute values of the features for MOVE and REST and healthy and impaired facial side, as well as an asymmetry index (AI), were determined. The AI was calculated as follows (shown using the MAV as sEMG feature), where 0% describes complete symmetry and a higher AI value describes increasing asymmetry between the facial sides:

$${AI}_{MAV}=\frac{\left({MAV}_{healthy}-{MAV}_{lesioned}\right)}{\left({MAV}_{healthy}+{MAV}_{lesioned}\right)}\times 100$$

### 2.4. Statistics

All statistical tests were performed using MATLAB (MathWorks Inc., Natick, MA, USA, R2022b) and SPSS (IBM SPSS Statistics for Windows, Version 30.0. Armonk, NY, USA, IBM Corp.). Univariate repeated-measure ANOVAs (rmANOVA) were performed separately for every movement type (i.e., smile strongly, smile lightly, close eyes strongly, close eyes lightly, raise forehead strongly, and raise forehead lightly) based on the absolute values of the time series feature to determine whether there is an effect of the state of motion (MOTION: MOVE vs. REST), side of the face (SIDE: healthy vs. lesioned), and/or House–Brackmann grade (HB) for each of the 20 features. MOTION and SIDE are between-subject factors, while HB was included as a within-subject factor. To account for the anatomical specificity of each facial movement, only electrodes relevant to the respective movement were included in the analysis: e.g., for mouth movements, electrodes positioned around the mouth were analyzed. The Mauchly test was performed to check the sphericity assumption and, if necessary, the degrees of freedom were adjusted using Greenhouse–Geisser correction.

Being the only between-subject factor, the statistical analysis of HB might be affected by the fact that time series values show a huge inter-subject variability. Absolute time series values are hardly comparable between subjects and depend on several other factors such as positioning of the electrodes, impedances, patient’s motivations. One widely used method to enable an inter-subject comparison is to normalize time series features to the healthy side resulting in the presented AI. Therefore, in addition to the rmANOVA, a two-tailed Spearman correlation analysis was performed between the AI of the 20 sEMG features and the HB score (i.e., HB I healthy controls, HB I patients, HB II + III, HB IV + V, HB VI). Furthermore, the Fisher Score (FS) for the AI of the features was calculated to assess the discriminative power of the features with respect to the different HB grades. The Fisher Score is defined as:

$$FS(f)=\frac{\sum_{i=1}^{C}n_{i}{\left(\mu_{i}-\mu \right)}^{2}}{\sum_{i=1}^{C}n_{i}\sigma_{i}^{2}}$$

where C is the number of classes (i.e., HB grades), $n_{i}$ is the number of samples in class i, $\mu_{i}$ is the mean of class i, μ is the overall mean of the feature across all samples, and $\sigma_{i}^{2}$ is the variance within class i. This score quantifies the relationship between within- and between-class variance, providing an additional measure of feature relevance for classification. Values of *p* < 0.05 were considered significant.

## 3. Results

### 3.1. Experimental and Clinical Characteristics

This study enrolled 55 subjects (51.2 ± 14.73 years; 35 female), including 40 patients with facial palsy HB grade II-VI, while 15 subjects had no facial palsy (HB I). The majority of FP were iatrogenic (37/40, 92.5%). On average, FP had been present for 1.84 years, and 8/55 (14.5%) subjects presented with facial aberrant reinnervation syndrome. All patients completed the study, and there were no side effects or undesired events during measurements. Specific cohorts’ characteristics are shown in Table 1.

### 3.2. Parameters for Motion Classification and Biofeedback Applications

**Figure 2:**
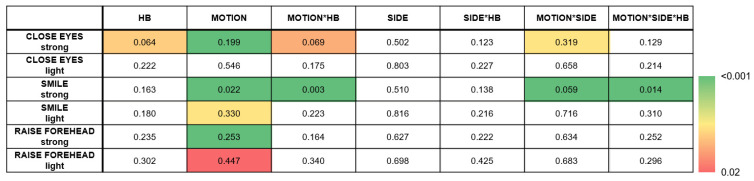
Multivariate test of repeated-measurement analysis showing the strongest effect for MOTION. While the box colors represent the significance level (*p*-value), the number values represent the Wilks’ λ. White boxes represent non-significant values.

**Figure 3:**
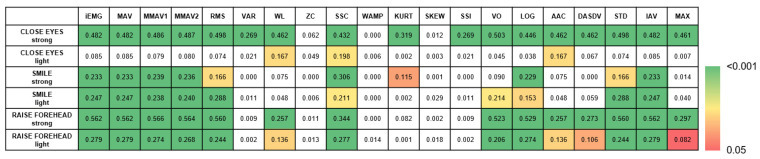
Univariate follow-up tests of MOTION in the rmANOVA. While the box colors represent the significance level (*p*-value), the number values represent the effect size (partial eta-squared, η^2^). White boxes represent non-significant values.

**Figure 4:**
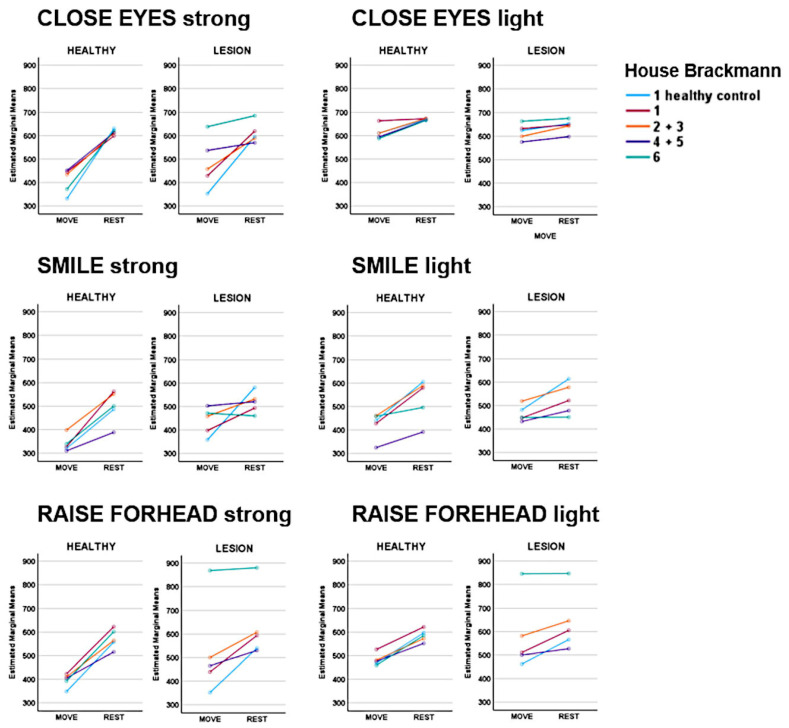
Slope sign change (SSC) values across different House–Brackmann (HB) scores. While MOVE and REST can be well differentiated on the healthy side by the SSC, the difference between MOVE and REST on the affected side decreases with increasing HB grade. However, distinctions can still be made up to HB grades IV + V.

The rmANOVA demonstrated a significant main effect of MOTION (MOVE/REST) for nearly all movement types, with the strongest effect apparent for strong movements (*p* < 0.001; Figure 2). Post hoc analysis revealed the slope sign change (SSC) as the best predictor of MOTION, although several other time series features revealed comparable performances: integrated EMG (iEMG), mean absolute value (MAV), modified mean absolute value 1 (MMAV1), modified mean absolute value 2 (MMAV2), root mean square (RMS), log detector (LOG), standard deviation (STD), and the integral of absolute value (IAV) (Figure 3). A detailed evaluation of the SSC showed good MOTION discrimination for the healthy SIDE. The difference between MOVE and REST on the affected side decreased with increasing HB grade. However, distinctions can still be made up to HB grades VI and V (Figure 4).

For the side of the face (SIDE), no significant effect was found for any of the movements, which can be attributed to the circumstance that (i) healthy subjects are also integrated in the analysis and (ii) there should be no difference between the two sides during REST. A significant effect for the HB was observed only in “CLOSE EYES strong” (Wilks’ Λ = 0.064, F(60, 114) = 1.77, *p* = 0.005). Post hoc tests revealed a significant result for all EMG features except zero crossing (ZC), slope sign change (SSC), Willison amplitude (WA), kurtosis (KURT) and skewness (SKEW).

In summary, sEMG features can discriminate MOTION independent for both sides lesioned and healthy (MOTION). However, only in the “CLOSE EYES strong” and “SMILE strong” conditions does sEMG detect differences between the SIDES, namely for the MOVE periode (MOTIONxSIDE). The HB affects the sEMG of the lesioned SIDE during SIDE but not REST (MOTIONxSIDExHB). However, this was only significant for the “SMILE strong” condition. Notably, unilateral FP seemed to also affect the sEMG of the healthy side (see HB, MOTIONxHB in “CLOSE EYES strong” and “SMILE strong” conditions).

### 3.3. Feature Extraction for Facial Nerve Grading

**Figure 5:**
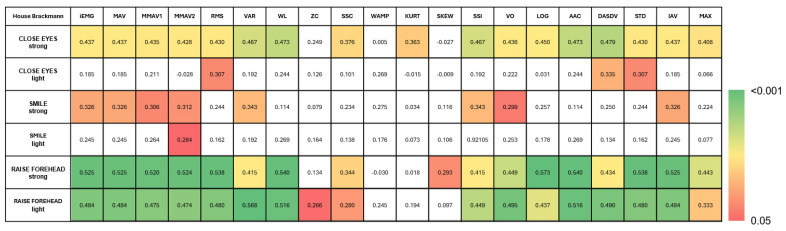
Correlation Heatmap demonstrates the correlation between the asymmetry index (AI) of the 20 time series features during different movements with the House–Brackmann score. While the box colors represent the significance level (*p*-value), the number values represent the strength of correlation (correlation coefficient). White boxes represent non-significant values.

**Figure 6:**
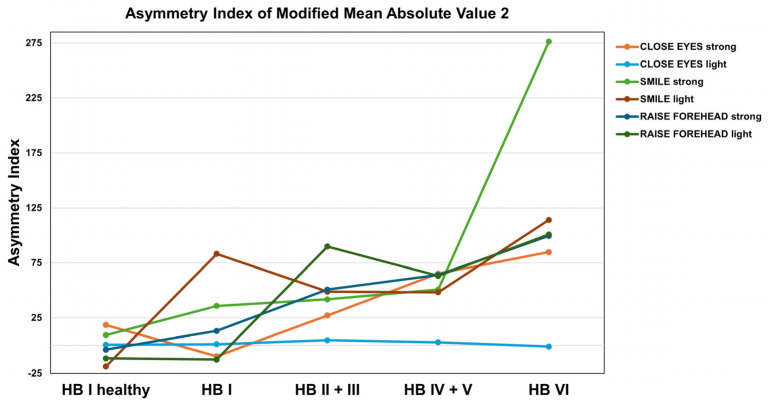
Asymmetry index of the modified mean absolute value 2 (MMAV2) demonstrating strongest correlations in forehead movements.

**Figure 7:**
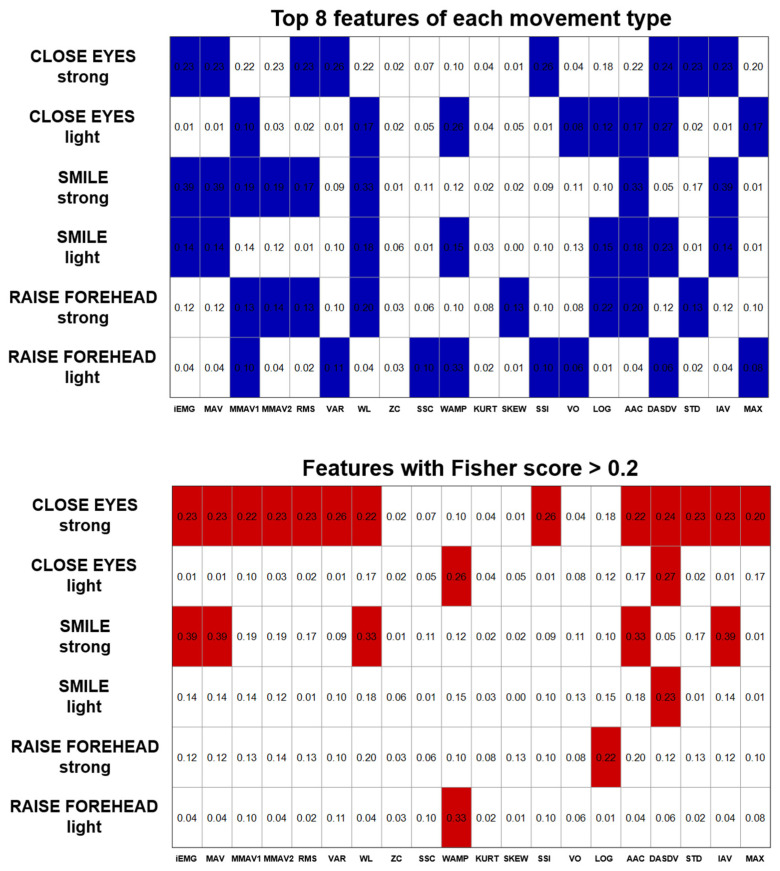
Fisher scores demonstrating the discriminative power of the AI of the EMG features with respect to the different HB grades. The top graph shows the 8 best features for each movement type, while the bottom graph highlights the Fisher scores >0.2.

Correlation analysis of the AI with the HB score indicated that only a few of the time series features indicated a significant correlation for the movements “CLOSE EYES light” and “SMILE light” (Figure 5). However, the MMAV2-AI correlated with the HB grade in 5/6 movement types (Figure 6). Also, iEMG, MAV, MMAV1, RMS, VAR, SSI, VO, DASDV, STD, and IAV correlated in four out of six movements. The strongest correlations were found for the forehead movements. In contrast, the forehead movements showed rather low Fisher scores (Figure 7). However, consistent with the correlation analysis, ZC, KURT, SSC and SKEW were found to discriminate poorly between the HB grades, while iEMG, MAV, DASDV and IAV appeared to be suitable for this purpose.

## 4. Discussion

Previous EMG biofeedback studies in FP are limited by utilizing feedback devices designed for extremities, although the fine mimic muscles of the face are structured differently than the muscles of the limbs [31]. In the present study, we were able to show that there are considerable differences between the various EMG time series features in the correlation to HB grade as well as in the classification between a movement and rest phase of the facial muscles. Future studies should therefore take into account that the facial EMG must be analyzed in a specialized manner.

Rutkowska et al. [28] identified in studies of emotional expressions the most frequently used features were the MAV, RMS and Integrated EMG (iEMG). In this context, our study shows the slope sign change to be suitable for distinguishing between different motion states (MOVE and REST). However, the SSC asymmetry index was not well correlated with the HB score in all movement types and intensities. In addition, the Fisher score analysis did not prove the SSC-AI to be suitable for HB classification. Combining statistical analyses (i.e., repeated measures, correlation analysis and Fisher score), iEMG, MAV, MMAV1, RMS and IAV seem to be the most proper parameters for the presented requirements, which is in line with the results of Rutkowska et al. [28]. These techniques all quantify the EMG’s energy information using mathematical calculations such as integration, averaging and squaring [26,27]. While the iEMG represents the summed activity of the EMG signal, MAV provides the mean absolute value. MMAV1 is a modified version of MAV to improve signal assessment depending on the specific measurement conditions. Whereas the RMS describes the signal energy and is more sensitive to high amplitudes, the IAV calculates the total absolute amplitude of the surface EMG signal over a given time period, providing a measure of the overall level of muscle activity regardless of signal polarity.

These parameters should be investigated further in future studies on sEMG and biofeedback in facial palsy. The main concern in this respect is to determine whether the combination of parameters or even the use of artificial intelligence with automated signal analysis might lead to better results than using only one parameter. This question is particularly important regarding (i) the classification of the severity of a facial palsy and (ii) the prediction of facial nerve recovery. While there are numerous studies investigating automatic FP classification using facial images and videos [32,33,34,35], there are only few studies using electrophysiological recordings (such as EMG). In this context, Holze et al. developed a semi-automated assessment system for facial nerve function based on sEMG and machine learning to replace the subjective HB score with a more objective method [12]. The sEMG showed promising performance (area under the curve [AUC]: 0.72–0.91) as a reliable alternative to the HB score. Although the present study and that of Holze et al. [12] primarily included patients with mild to moderate FP (HB I–III), several noteworthy methodological and clinical differences distinguish the two. We included a larger cohort and focused not only on FP severity classification but also on identifying interpretable and robust EMG parameters that could serve as the foundation for future biofeedback training. Therefore, EMG signals were analyzed not only during active movement, but also during rest phases to investigate whether the parameters can differentiate between the two states. This distinction is particularly relevant for biofeedback applications, where dynamic changes and asymmetries between rest and activation play a central role. Furthermore, while the machine learning approach by Holze et al. [12] was optimized for classification accuracy, such models may be less suited for biofeedback purposes, where transparent and user-understandable feedback parameters are needed to guide patient engagement and training effectiveness.

Most studies on prediction models for FP focus on the probability of predicting the occurrence of FP after certain procedures (e.g., resection of vestibular schwannomas) and/or the use of clinical parameters (e.g., size of tumors, age of a patient, severity of paresis) [36,37,38,39]. There was only one study by Khisimoto et al. that investigated the predictive value of different artificial intelligence models for predicting the occurrence of synkinesia after FP using electroneurography [40]. The results demonstrated a good predictive performance (AUC: 0.90.) of a machine-learning-based logistic regression method, which represents a contrast-or rather progress-compared to a study by Azuma et al. [41], who were unable to predict the occurrence of synkinesia using the ENoG (without using machine learning). However, we are not aware of any studies that combine sEMG and artificial intelligence for prediction of FP outcome in this context.

Further factors to consider when evaluating sEMG for facial biofeedback are the stages of nerve damage and recovery. As in the case of a peripheral nerve injury of the limbs, various degrees of nerve injury (i.e., neuropraxia, axonotmesis, neurotmesis) can also occur in peripheral FP [42,43,44]. The extent of clinical impairment depends on this, as does the speed of recovery. EMG biofeedback, therefore, must differ during the paralytic phase than during the synkinetic phase of a FARS. However, a common point to emphasize is that the aim of facial training-such as neuromuscular retraining-is not to develop maximum muscle contraction, as this tends to promote syn- and dyskinesia (even in the paralytic phase) [45]. Instead, the aim is to learn to control the force intensity and coordinate the facial muscles. Our study addresses this by examining the three movement types, and thus the usefulness of the time series features, for different contraction strengths (strong and light). The results indicate that some time series features, such as kurtosis or waveform length, could be used for the stronger movements, but may not be able to adequately detect differences between movement and rest for weaker movements. Hence, in early phases or more severe paresis, robust features such as RMS and MAV may be preferable, whereas in later phases—when the aim is to improve coordination of the mimic muscles and reduce synkinesia—other parameters may be more suitable. A stage-specific selection of features could improve the effectiveness of facial EMG biofeedback by aligning signal analysis with the functional needs of each recovery phase. However, our study only controlled the different intensities using instructions. In the future, it would be conceivable to control the intensity objectively, e.g., by using the sEMG of the healthy side, by grading the intensity as a percentage of the maximum force or by combining the sEMG with kinematic measurements or visual controls [46,47].

### Limitations

The main limitation of the study is that the EMG features were only examined using 12 electrodes (6 per facial side, bipolar setting). However, the mimic musculature consists of >15 muscles, so interference phenomena and inaccuracies may occur due to our electrode setup. More precise statements about muscle function might be possible using a high-density EMG [48,49,50,51]. At the same time, the many electrodes may contribute to reduced mobility in the facial area. This would be contrary to a biofeedback application. Another limitation lies in the unbalanced cohort, with only 7 healthy controls compared to 48 patients. However, the focus of the study was not a direct statistical comparison between healthy and patient groups, but rather the evaluation of suitable time series features of an EMG for biofeedback training and the classification of FP. The uneven distribution across HB grades also reflects typical clinical reality but limits the generalizability of subgroup comparisons. Future studies with larger and more balanced samples are warranted to validate the findings. Finally, other important factors influencing the outcome of biofeedback interventions—such as training frequency, session duration, or the specific type of feedback (e.g., visual, auditory, multimodal)—were not within the scope of our investigation. These parameters, however, likely have a considerable impact on training efficacy and user compliance and should be systematically explored in future studies. Integrating these aspects into experimental designs may help to better understand how to tailor biofeedback interventions for different stages of FP and individual patient needs.

## 5. Conclusions

The research findings indicate that sEMG can reliably determine facial palsy severity and guide biofeedback interventions after facial palsy. Appropriate EMG parameters, such as the iEMG, RMS or MAV, should be used to enable the best possible differentiation between rest and movement, even with minor movements and severe paresis. In this context, the present study constitutes the foundation for further investigations of biofeedback algorithms and training modalities required for EMG biofeedback in facial palsy.

## Figures and Tables

**Table 1:** Cohort characteristics.

	Total *n* = 55	Patients *n* = 48	Healthy Subjects *n* = 7
Age	51.2 ± 14.73	53.85 ± 12.62	32.57 ± 15.7
Gender			
male	20 (36.4%)	17 (35.4%)	3 (42.9%)
female	35 (63.6%)	31 (64.6%)	4 (57.1%)
HB grade			
I	15 (27.2%)	8 (16.7%)	7 (100%)
II	8 (14.5%)	8 (16.7%)	0 (0%)
III	21 (38.2%)	21 (43.8%)	0 (0%)
IV	5 (9.1%)	5 (10.4%)	0 (0%)
V	4 (7.3%)	4 (8.3%)	0 (0%)
VI	2 (3.6%)	2 (4.2%)	0 (0%)
Side of FP/or surgery			
right	19 (34.5%)	19 (34.5%)	0 (0%)
left	29 (52.7%)	29 (52.7%)	0 (0%)
no facial palsy/surgery	7 (12.7%)	0 (0%)	7 (100%)
Etiology of FP			
idiopathic	2 (3.6%)	2 (4.2%)	0 (0%)
tumor	1 (1.8%)	1 (2.1%)	0 (0%)
iatrogenic	37 (67.3%)	37 (77.1%)	0 (0%)
no FP	15 (27.3%)	8 (16.6%)	7 (100%)
Period since onset of FP/surgery			-
overall	672.27 ± 2158.78 days (=1.84 years)	672.27 ± 2158.78 days (=1.84 years)	
HB I	3.25 ± 1.04 days	3.25 ± 1.04 days	
HB II-III	850.97 ± 2431.85 days	850.97 ± 2431.85 days	
HB IV-V	839.44 ± 2444.37 days	839.44 ± 2444.37 days	
HB VI	5.00 ± 1.41 days	5.00 ± 1.41 days	
FARS			
yes	8 (14.5%)	8 (16.7%)	0 (0%)
no	47 (85.5%)	40 (83.3%)	7 (100%)

HB: House–Brackmann score; FP: facial palsy; FARS: facial aberrant reinnervation syndrome; surgery always means posterior skull base surgery (i.e., VS surgery or meningioma).

## Data Availability

The data presented in this study are available on request from the corresponding author due to ethical reasons.

## Abbreviations

The following abbreviations are used in this manuscript:

AAC	average amplitude change
AI	asymmetry index
AUC	area-under-the-curve
DASDV	difference absolute standard value
FARS	facial aberrant reinnervation syndrome
FP	facial palsy
HB	House–Brackmann score
IAV	integral of absolute value
iEMG	intregrated electromyography
KURT	kurtosis
LOG	log detector
MAV	mean absolute value
MAX	maximal amplitude
MMAV1	modified mean absolute value 1
MMAV2	modified mean absolute value 2
QoL	quality of life
RMS	root mean square
sEMG	surface electromyography
SKEW	skewness
SSC	slope sign change
SSI	simple square integral
STD	standard deviation
VAR	variance
VO	V-order
VS	vestibular schwannoma
WAMP	Willison amplitude
WL	waveform length

## Appendix A

**Table A1:** Description of time series features.

Feature	Abbrev.	Mathematical Definition		Description
Integrated EMG	iEMG	$iEMG=\sum_{i=1}^{N}\left|x_{i}\right|$	N = length of signal i = EMG signal in a segment	Sum of the absolute values of the signal in a sliding window Robust against small fluctuations in the EMG signal, as it considers the whole signal
Mean Absolute Value	MAV	$MAV=\frac{1}{N}\sum_{i=1}^{N}\left|x_{i}\right|$	N = length of signal i = EMG signal in a segment	Mean of the absolute values of the signal in a sliding window; calculated by summing the absolute values of the EMG signal and dividing them by the number of signal points
Modified Mean Absolute Value 1	MMAV1	$MMAV1=\frac{1}{N}\sum_{i=1}^{N}w_{i}\left|x_{i}\right|$ $w_{i}=\left\{\begin{array}{c} 1,\;if\;0.25\;N\leq i\leq 0.75\;N\\ 0.5,\;otherwise \end{array}\right.$	N = length of signal $x_{i}$ = EMG signal in a segment $w_{i}$ = weighting function that determines the position within the time window	all values of the EMG signal are considered but values at the edge of the time window are weighted more strongly than values in the middle
Modified Mean Absolute Value 2	MMAV2	$MMAV2=\frac{1}{N}\sum_{i=1}^{N}w_{i}\left|x_{i}\right|$ $w_{i}=\left\{\begin{array}{c} 1,\;\;if\;0.25\;N\leq n\leq 0.75\;N\\ \frac{4n}{n},\;if\;n<0.25N\\ \frac{4(N-n)}{N},\;\;otherwise \end{array}\right.$	N = length of signal $x_{i}$ = EMG signal in a segment $w_{i}$ = weighting function that determines the position within the time window	all values of the EMG signal are considered but values at the middle are weighted more strongly than values at the edge of the time window
Root Mean Square	RMS	$RMS=\sqrt{\frac{1}{N}}\sum_{i=1}^{N}{x_{i}}^{2}$	N = length of signal $x_{i}$ = EMG signal in a segment	square root of the average of the EMG amplitude in a sliding window Wahl des Analysefensters beeinflusst RMS deutlich
Variance	VAR	$VAR=\frac{1}{N-1}\sum_{i=1}^{N}{x_{i}}^{2}$	N = length of signal $x_{i}$ = EMG signal in a segment	measures the variation of the amplitude values of the EMG signal compared to its mean value provides information about the consistency of a signal in a certain period of time
Waveform Length	WL	$WL=\sum_{i=1}^{N}\left|x_{i+1}-x_{i}\right|$	N = length of signal $x_{i}$ = EMG signal in a segment	average distance between two consecutive zero crossings in the EMG signal Schwankung Muskelaktivität im Zeitverlauf → z.B. gut zur Analyse von Muskelermüdung anwendbar
Zero Crossing	ZC	$ZC=\sum_{i=1}^{N}f\left(x\right)$ $f\left(x\right)=\left\{\begin{array}{c} 1,\;if\;x_{i}\times x_{i+1}<0\;and\;\left|x_{i}-x_{i+1}\right|\geq threshold\;\\ 0,\;otherwise \end{array}\right.$	N = length of signal $x_{i}$ = EMG signal in a segment	number of times that the EMG signal crosses the zero value in each time interval indicator for the direction and frequency of changes in muscle activity
Slope Sign Change	SSC	$SSC=\sum_{i=2}^{N}f\left[\left(x_{i}-x_{i-1}\right)\times \left(x_{i}-x_{i+1}\right)\right]$ $f\left(x\right)=\left\{\begin{array}{c} 1,\;if\;x\;\geq threshold\;\\ 0,\;otherwise \end{array}\right.$	N = length of signal $x_{i}$ = EMG signal in a segment	point at which the slope of the EMG signal changes from positive to negative or from negative to positive
Willison Amplitude	WAMP	$WAMP=\sum_{i=1}^{N-1}f\left(\left|x_{i}-x_{i+1}\right|\right)$ $f\left(x\right)=\left\{\begin{array}{c} 1,\;if\;x\geq treshold\\ 0,\;\;otherwise \end{array}\right.$	N = length of signal $x_{i}$ = EMG signal in a segment Threshold in present study: 10% of the maximum absolute amplitude of the signal	Sum of the amplitude changes in the EMG signal that exceed a certain threshold within a sliding window
Kurtosis	KURT	$KURT=\frac{1}{N}\sum_{i=1}^{N}{\left(\frac{x_{i}-x¯}{\sigma }\right)}^{4}$	N = length of signal $x_{i}$ = EMG signal in a segment $\bar{x}$ = mean of the data σ = standard deviation of dataset	measures the shape of the amplitude distribution of the EMG signal. It indicates whether the distribution is relatively peaked (more values in the center) or more flat-topped (more values in the tails)
Skewness	SKEW	$SKEW=\frac{1}{N}\sum_{i=1}^{N}{\left(\frac{x_{i}-x¯}{\sigma }\right)}^{3}$	N = length of signal/number of signal values $x_{i}$ = EMG signal at i $\bar{x}$ = mean of the data σ = standard deviation of signal	describes the degree of asymmetry of a distribution around ist mean
Simple Square Integral	SSI	$SSI=\sum_{i=1}^{N}{\left|x_{i}\right|}^{2}$	N = length of signal $x_{i}$ = EMG signal in a segment	represents the energy of the EMG signal and is often defined as energy index of the EMG signal
V-Order	VO	$V={\left(\frac{1}{N}\sum_{i=1}^{N}x_{i}^{v}\right)}^{\frac{1}{v}}$	N = length of signal/number of signal values $x_{i}$ = EMG signal at i v = order of calculation (in present study 3)	feature that describes the distribution and fluctuations of the signal strengths in an EMG by summarizing the amplitudes in a certain power v
Log detector	LOG	$LOG=e^{\frac{1}{N}\sum_{i=1}^{N}log\left|x_{i}\right|}$	N = length of signal $x_{i}$ = EMG signal in a segment	provides an estimate of the muscle contraction force by using a logarithmic function helps to better visualize differences in amplitudes, which is advantageous for smaller muscle activities that would otherwise be difficult to distinguish
Average Amplitude Change	AAC	$AAC=\frac{1}{N}\sum_{i=1}^{N-1}\left|x_{i+1}-x_{i}\right|$	N = length of signal $x_{i}$ = EMG signal in a segment	nearly equivalent to WL feature, except that wavelength is averaged high AAC indicates that muscle activity fluctuates strongly over time
Difference Absolute Standard Value	DASDV	$DASDV=\sqrt{\frac{1}{N-1}\sum_{i=1}^{N-1}{\left(x_{i+1}-x_{i}\right)}^{2}}$	N = length of signal $x_{i}$ = EMG signal in a segment	looks like a combination of RMS and WL, which can be seen as the standard deviation of the wavelength
Standard Deviation	STD	$STD=\sqrt{\frac{1}{N}\sum_{i=1}^{N}{\left(x_{i}-x¯\right)}^{2}}$	N = length of signal $x_{i}$ = EMG signal in a segment $\bar{x}$ = mean value of the EMG signal	difference between each sample of EMG and its mean value
Integral of Absolute Value	IAV	$IAV=\sum_{i=1}^{N}\left|x_{i}\right|$	N = length of signal/number of samples $x_{i}$ = EMG signal at instance i	absolute average value of each sample or segment
Maximal Amplitude	MAX	$MAX=max\;(\left|x_{i}\right|)$	$x_{i}$ = EMG signal as function of time max = maximum function that determines the largest value from the signal amounts	largest value measured in the EMG signal

(Chowdhury et al., 2013 [27]; Nazmi et al., 2016 [26]).
